# Deciphering Structural Alterations Associated with Activity Reductions of Genetic Polymorphisms in Cytochrome P450 2A6 Using Molecular Dynamics Simulations

**DOI:** 10.3390/ijms221810119

**Published:** 2021-09-19

**Authors:** Koichi Kato, Tomoki Nakayoshi, Rika Nokura, Hiroki Hosono, Masahiro Hiratsuka, Yoshinobu Ishikawa, Eiji Kurimoto, Akifumi Oda

**Affiliations:** 1 Faculty of Pharmacy, Meijo University, 150 Yagotoyama, Tempaku-ku, Nagoya 468-8503, Japan; kato-k@kinjo-u.ac.jp (K.K.); nakayoshi@hiroshima-cu.ac.jp (T.N.); 140973350@ccalumni.meijo-u.ac.jp (R.N.); kurimoto@meijo-u.ac.jp (E.K.); 2 Faculty of Pharmaceutical Sciences, Shonan University of Medical Sciences, 16-48 Kamishinano, Totsuka-ku, Yokohama 244-0806, Japan; yoshinobu.ishikawa@sums.ac.jp; 3 College of Pharmacy, Kinjo Gakuin University, 2-1723 Omori, Moriyama-ku, Nagoya 463-8521, Japan; 4 Graduate School of Information Sciences, Hiroshima City University, 3-4-1 Ozukahigasi, Asaminami-ku, Hiroshima 731-3194, Japan; 5 Graduate School of Pharmaceutical Sciences, Tohoku University, Sendai 980-8578, Japan; hosono.h.c2@hosp.tohoku.ac.jp (H.H.); masahiro.hiratsuka.a8@tohoku.ac.jp (M.H.); 6 Department of Pharmaceutical Sciences, Tohoku University Hospital, Sendai 980-8574, Japan; 7 Tohoku Medical Megabank Organization, Tohoku University, Sendai 980-8573, Japan; 8 Advanced Research Center for Innovations in Next-Generation Medicine, Tohoku University, Sendai 980-8573, Japan; 9 Institute for Protein Research, Osaka University, 3-2 Yamadaoka, Suita 565-0871, Japan

**Keywords:** cytochrome P450, drug-metabolizing enzyme, molecular dynamics simulation, genetic polymorphism, structural analysis

## Abstract

Cytochrome P450 (CYP) 2A6 is a monooxygenase involved in the metabolism of various endogenous and exogenous chemicals, such as nicotine and therapeutic drugs. The genetic polymorphisms in CYP2A6 are a cause of individual variation in smoking behavior and drug toxicities. The enzymatic activities of the allelic variants of CYP2A6 were analyzed in previous studies. However, the three-dimensional structures of the mutants were not investigated, and the mechanisms underlying activity reduction remain unknown. In this study, to investigate the structural changes involved in the reduction in enzymatic activities, we performed molecular dynamics simulations for ten allelic mutants of CYP2A6. For the calculated wild type structure, no significant structural changes were observed in comparison with the experimental structure. On the other hand, the mutations affected the interaction with heme, substrates, and the redox partner. In CYP2A6.44, a structural change in the substrate access channel was also observed. Those structural effects could explain the alteration of enzymatic activity caused by the mutations. The results of simulations provide useful information regarding the relationship between genotype and phenotype.

## 1. Introduction

Cytochrome P450 (CYP) is a monooxygenase that plays a central role in the synthesis pathways of steroids, fatty acids, lipid-soluble vitamins, and eicosanoids [1]. In addition, CYP is a primary enzyme for drug metabolism [2,3,4]. The active site of CYP includes an heme iron that mediates electron transfer related to oxidation reactions [5,6]. Drug metabolism by CYP proceeds via oxidation, reduction (phase I), hydrolysis, and conjugation (phase II). The phase I oxidation requires electron carriers such as nicotinamide adenine dinucleotide phosphate (NADPH), and electron transfers are mediated by the membrane-bound flavor protein CYP redox partner [7]. The binding of CYP and the redox partner is necessary for electron transfer, and the helices C, K, and L of CYP are predicted to interact with the redox partner [8,9,10,11].

**Figure 1:**
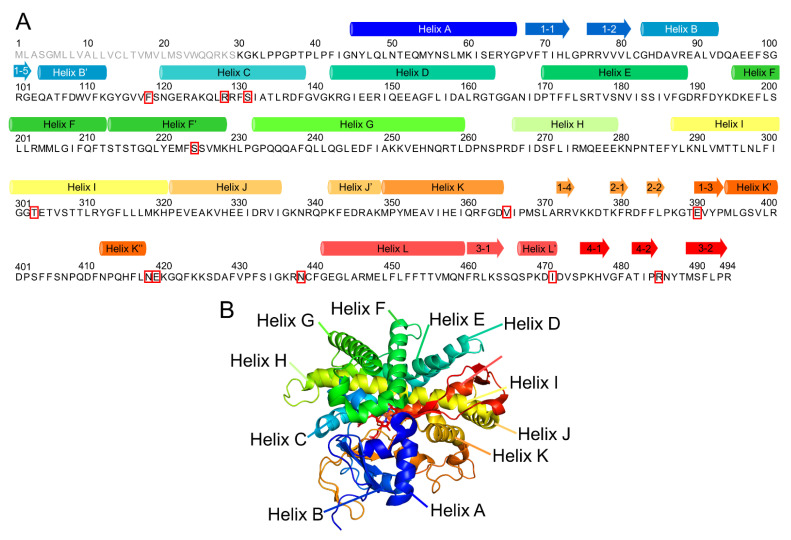
Numbering of the secondary structures of CYP2A6. (**A**) Correspondence between the amino acid sequence and secondary structures. The circular column and arrow indicate helix and β-strand formation regions, respectively. The gray letters of the amino acid sequence are the disordered region in the crystal structure. The mutation sites relevant to 10 variants investigated in this study are shown in red squares. (**B**) Correspondence between the three-dimensional structure and secondary structures.

One of the members of CYP, CYP2A6, is involved in the metabolism of nicotine and various therapeutic drugs such as tegafur, valproic acid, pilocarpine, propofol, and cisapride [12,13]. In addition, Jiang et al. reported that CYP2A6 was a protective factor of hepatocellular carcinoma [14]. The cavity in the active site of CYP2A6 is about 25% of the volume of those in CYP2C8, CYP2C9, and CYP3A4 [15]. Therefore, the substrates and inhibitors for CYP2A6 are relatively small molecules [16,17]. The amino acid sequence and whole structure of CYP2A6 is shown in Figure 1. The structure includes the helices A–L and β1–4. CYP2A6 has six substrate-recognizing sites (SRS-1–6) [18]. SRS-1 is composed of B’ and a flanking area (residues 102–112), SRS-2 is composed of the C-terminal end of helix F (residues 197–204), SRS-3 is composed of the N-terminal end of helix G (residues 232–239), SRS-4 is composed of the N-terminal half of helix I (residues 288–296), SRS-5 is composed of the β2 area (residues 358–368), and SRS-6 is composed of the central region of β5 (residue 468–476). The flexibilities of helices F and G, including SRS-2 and SRS-3, respectively, are markedly reduced by substrate binding. Therefore, these helices are considered important for the accommodation of enzymatic activities.

The *CYP2A6* gene is mapped to chromosome 19 and located in a gene cluster composed of 350 kbp, together with the *CYP2A7*, *CYP2A13*, and *CYP2B* subfamilies, and the CYP*2F* subfamily [13]. *CYP2A7* is known as a nonfunctional pseudogene. Phenotyping and in vitro studies indicate the prevalence of *CYP2A6* polymorphisms. Although the CYP2A6 poor metabolizers are <1% of the Caucasian population, they comprise 20% of the Orientals population [19,20]. The presence of *CYP2A6* polymorphisms is a risk factor for drug toxicity and tobacco-related lung cancer in male Japanese smokers [21]. Several *CYP2A6* polymorphisms are characterized. CYP2A6*3, CYP2A6*4, CYP2A6*12, CYP2A6*20, CYP2A6*27, and CYP2A6*34 are inactive because of frame-shift mutations, gene conversion with the pseudogene *CYP2A7*, or whole gene deletion [22,23]. In CYP2A6*9, a point mutation in the TATA box located in the 5′ flanking region reduces the transcriptional level. CYP2A6*29, CYP2A6*30, CYP2A6*32, and CYP2A6*33 alleles are still being evaluated. Otherwise, 34 CYP2A6 mutants are characterized, and their metabolizing activities are evaluated by the assays for nicotine C-oxidization and coumarin 7-hydroxylation [22,24]. In CYP2A6*14 and CYP2A6*15, the nicotine and coumarin metabolizing activities are higher than those of the wild type. These activities of CYP2A6*28 and CYP2A6*31 are the same levels as those of the wild type. Other mutations reduce metabolizing activities. However, the detailed mechanisms of the metabolizing activity reductions are obscure because experimental structures of CYP2A6 mutants are unsolved.

In this study, to investigate the structures of CYP2A6 mutants, we performed molecular dynamics (MD) simulations for 10 mutants (CYP2A6.6, CYP2A6.11, CYP2A6.17, CYP2A6.25, CYP2A6.26, CYP2A6.35, CYP2A6.36, CYP2A6.37, CYP2A6.43, and CYP2A6.44). The selected mutants and their metabolizing activities are shown in Table 1. To assess the reason for metabolizing activity reduction, we selected variants with lower activities than the wild type. The allele frequencies of CYP2A6.6 and CYP2A6.11 were 0.4 and 0.6%, respectively, in healthy Japanese people [22,25]. CYP2A6.17 was frequently detected in African Americans (9.4%) [22]. Although the frequencies of CYP2A6.43 and CYP2A6.44 were lower (0.2% in African American) [26], the enzymatic activities were drastically lower than that of CYP2A6.17 (Table 1). The frequencies of CYP2A6.25 and CYP2A6.26 were 0.5 and 0.7%, respectively, in African Americans [27]. Those of CYP2A6.35 were 2.8% African Canadian and 0.8% Japanese [28]. CYP2A6.36 and CYP2A6.37 were detected in 0.3% of the Taiwanese population [28]. The MD simulations for CYP2A6.25, CYP2A6.26, CYP2A6.35, CYP2A6.36, and CYP2A6.37 were performed to investigate the effects of mutation combinations on three-dimensional (3D) structures. MD simulations were variable methods to investigate the structural features of mutant proteins including CYP [29,30,31,32,33,34]. For comparison, the simulation of the wild type (CYP2A6.1) was also performed.

## 2. Results and Discussion

**Figure 2:**
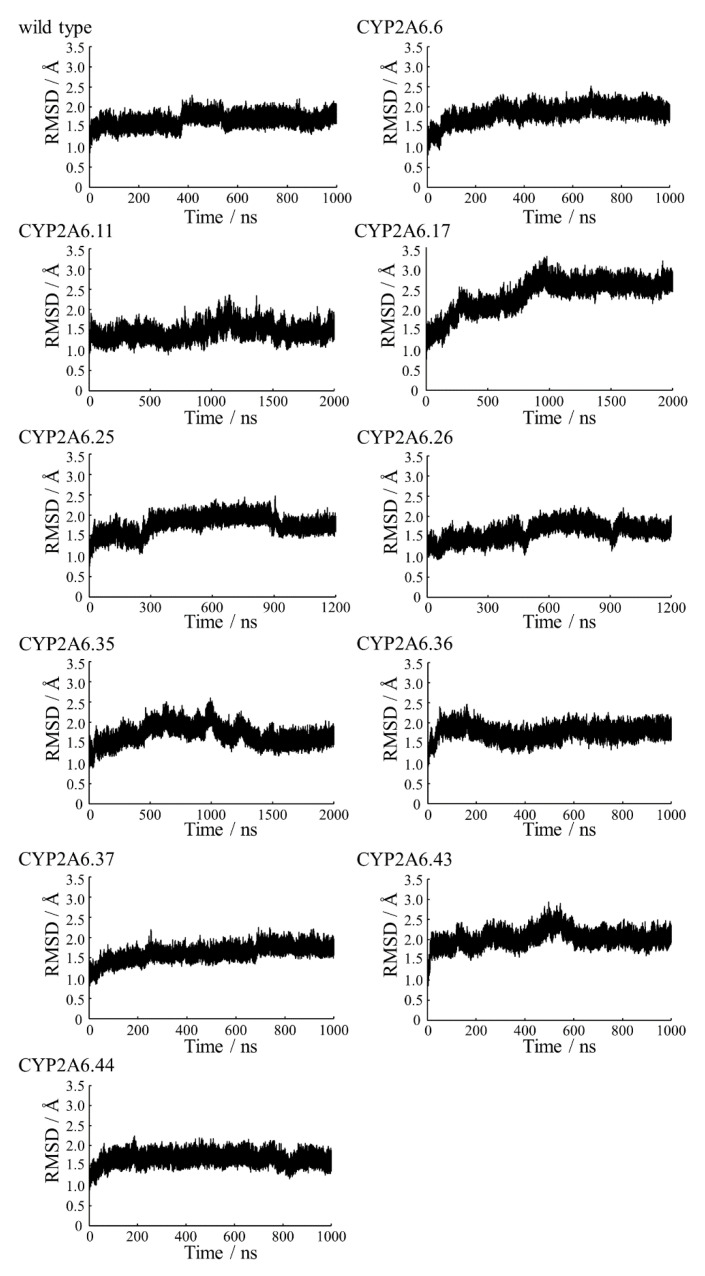
Root mean square deviations (RMSD) plots of the main-chain atoms for CYP2A6 wild type and mutants.

**Figure 3:**
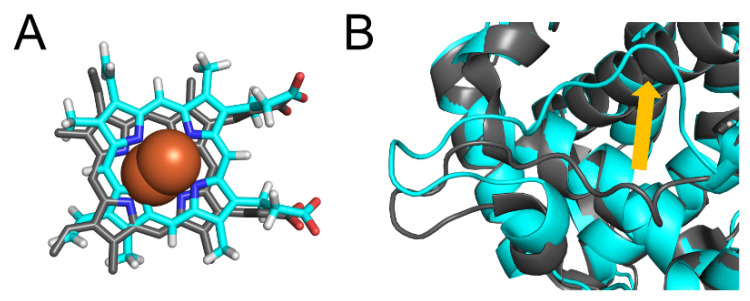
Structural deviation between the experimental and calculated structures. (**A**) The deviation of heme and (**B**) the K”L loop. The experimental structure and calculated structures are shown in gray and cyan, respectively. Nitrogen, oxygen, and hydrogen are displayed in blue, red, and white, respectively, in the stick model. Iron is shown as an orange sphere by a model written as van der Waals radius. The yellow arrow indicates the deviation of the K”L loop.

**Figure 4:**
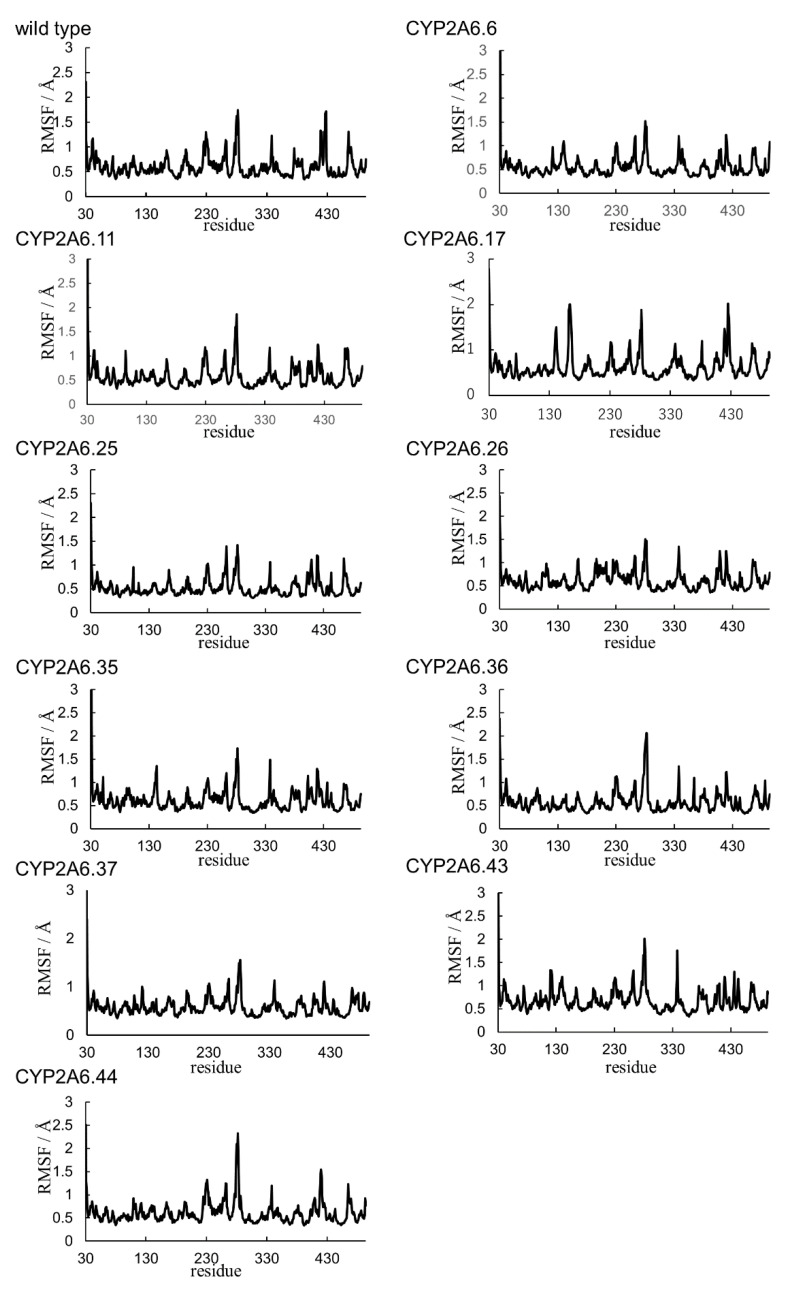
RMSF plots of Cα atoms in the last 10 ns of simulations.

To investigate the structures of CYP2A6 mutants, the MD simulations were performed, and the convergences were evaluated by RMSDs (Figure 2). Each calculation was converged during the simulation times. The final structures obtained by the simulations are shown in Figure S1. From the final structures and RMSDs, large structural collapses were not observed within the simulations for all mutants. In the wild type, the location of heme in the calculated structure was similar to that of the experimental structure (Figure 3A). In previous studies, short MD simulations for the wild type were performed [35,36]. In [36], the simulation converged within 20 ns and continued until 100 ns. The simulations of the wild type in this study seemed to converge within 100 ns; however, the RMSD was largely changed at around 400 ns. Therefore, the simulation of CYP2A6 needed to be over 400 ns. In this calculated structure, there was almost no deviation from the experimental structure, except for the K”L loop (Figure 3B). The root mean square fluctuation (RMSF) values of residues 427 and 428 in the K”L loop were relatively high (≥1.5 Å) (Figure 4). Therefore, the shifts in K”L loops in the final structure were likely due to their flexibility. The RMSF value of Pro282 in the HI-loop was 1.75 Å, but the flexibilities of other regions were low. The alterations of secondary structure formations are shown in Table 2 and Table 3. In CYP2A6.6, CYP2A6.26, CYP2A6.36, CYP2A6.37, and CYP2A6.44, no significant alterations were observed. Helices F’ and L were separated into two helices in CYP2A6.11. Helix I was separated into two helices in CYP2A6.25 and CYP2A6.43. In addition, helix L was separated into three helices in CYP2A6.17. The formation of β4 was impaired in CYP2A6.35.

### 2.1. CYP2A6.6 Affected the Interaction with the Redox Partner

**Figure 5:**
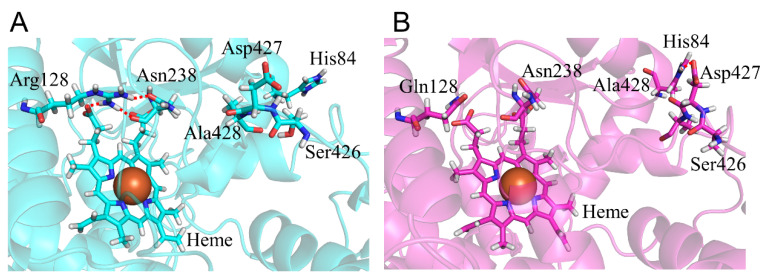
Structural change in CYP2A6.6. The wild type (**A**) and CYP2A6.6 (**B**) are shown in cyan and magenta, respectively. Nitrogen, oxygen, and hydrogen are displayed in blue, red, and white, respectively, in the stick model. Iron is shown as an orange sphere by a model written as the van der Waals radius. The red dotted lines indicate the hydrogen bonds.

To evaluate the structure of CYP2A6.6, we compared the calculated results of the R128Q mutant with those of the wild type. The catalytic activity of CYP2A6.6 for nicotine and coumarin was reportedly undetectable [22]. Although, for Arg128 locates on helix C, the helix structure was not collapsed by the R128Q mutation (Table 2). However, the length change in the side chain and the loss of the positive charge of residue 128 affected hydrogen bond formation and interaction with the K”L loop. In the wild type, Arg128 formed an hydrogen bond with heme (Table 4 and Figure 5). On the other hand, Gln128 of the R128Q mutant formed no hydrogen bond with heme. The R128Q mutation was considered to decrease heme-binding affinity. Arg128 formed hydrogen bonds with Asn438 located on the K”L loop in the wild type, but these were lost in R128Q mutants. The loss of the hydrogen bond formation affected the structure and flexibility of K”L loop. In CYP2A6.6, Ser426–Ala428 were close to helix B, and Asp427 formed the ionic bonds with His84. This ionic bond was not observed in the wild type. In addition, the RMSF values of Ser426-Ala428 were smaller than the wild type (Figure 4B). Therefore, R128Q mutation shifted the K”L loop to helix B and decreased the flexibility of the K”L loop. Those alterations in the K”L loop structure could affect the interaction with the CYP redox partner. For this interaction with redox partner, R128Q mutation on helix C could have a direct effect because arginine residues on helix C were reported to be important for the interaction with the redox partner [10]. Therefore, the hindrance of the complex formation with the redox partner was suggested to be one of the reasons for loss of function in CYP2A6.6.

### 2.2. CYP2A6.11 Affected Secondary Structure Formation and Interaction with Heme

**Figure 6:**
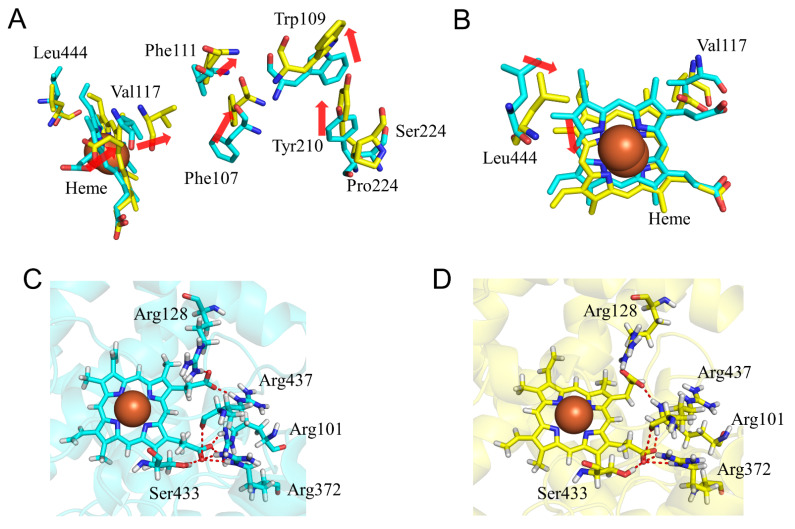
Structural change in CYP2A6.11 (**A**,**B**). The wild type (**C**) and CYP2A6.11 (**D**) are shown in cyan and yellow, respectively. In (**A**,**B**), all hydrogen atoms are omitted. Nitrogen, oxygen, and hydrogen are displayed in blue, red, and white, respectively, in the stick model. Iron is shown as an orange sphere by a model written as van der Waals radius. The red dotted lines indicate the hydrogen bonds. The red arrows indicate the shift in CYP2A6.11.

To evaluate the structural change in CYP2A6.11, the calculated results of the S224P mutant were compared with those of the wild type. The intrinsic clearances (*CL_int_*) for nicotine and coumarin in CYP2A6.11 were 18% and 26%, respectively, of those in the wild type [22]. Ser224 was included in helix F’, and the S224P mutation interrupted helix formation. Namely, helix F’ of CYP2A6.11 was separated into two helices composed of Ser215–Met222 and Pro224–Met227 (Table 2). The proline residing in an α-helix was known to cause a kink and the destabilization of the helical structure [37,38]. Therefore, the S224P mutation caused the separation of helix F’ by destabilizing the helical structure. In addition, helix L without the mutation was also separated into two helices composed of Gly441–Gly443 and Met447–Asn459. This alteration of helical structure was also caused by the effect of the S224P mutation. The structural change of helix F’ by S224P mutation altered the location of Tyr210 on helix F’ (Figure 6A). The shifted side chain of Tyr210 displaced Trp109 and helix B’, including this tryptophan residue. Val117 on the B’C loop moved into the space generated by the migration of Phe107 and Phe111 on helix B’. These phenylalanine residues were associated with the interaction with coumarin in the complex structure [15,39]. In addition, Val117 shifted the location of heme to the same direction as the shift of this valine residue. According to the deviation of the heme location, Leu444 on helix L was moved, and the helix L was separated (Figure 6B). The structural change in helix L could affect the interaction with the redox partner. The deviation of heme altered the hydrogen bond formation between heme and surrounding residues (Table 5). Although the wild type Arg101 formed an hydrogen bond only with O_A_, in the S224P mutant Arg101 formed hydrogen bonds with the oxygen of carbonic acid moiety, binding the pyrrole ring A (O_A_) and D (O_D_) of heme. Hydrogen bond formation between Arg372 and O_A_ was attenuated in the S224P mutant. We concluded that the catalytic activity of CYP2A6.11 decreased due to the deviation of heme caused by the changes in the helix B’ structure and the decrease in the binding affinity of the redox partner.

### 2.3. CYP2A6.17 Affected Secondary Structure Formation and Interaction with Heme and Redox Partoner

**Figure 7:**
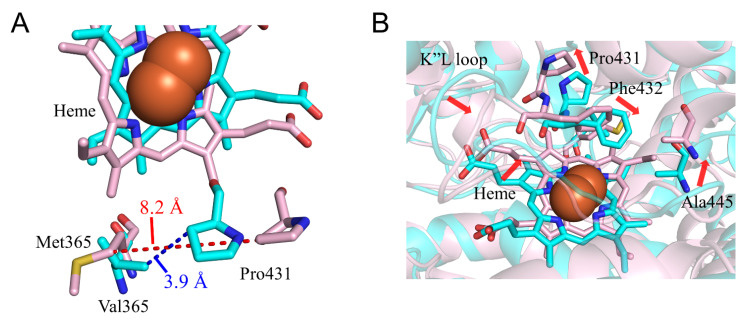
Structural change in CYP2A6.17 (**A**,**B**). The wild type and CYP2A6.17 are shown in cyan and pink, respectively. Nitrogen, oxygen, and hydrogen are displayed in blue, red, and white, respectively, in the stick model. Iron is shown as an orange sphere by a model written as van der Waals radius. The red and blue dotted lines indicate the distances between Val/Met365 and Pro431. The red arrows indicate the shift in CYP2A6.17. All hydrogen atoms are omitted.

To evaluate the structural change in CYP2A6.17, the calculated results for the V365M mutant were compared with the wild type. The *CL_int_* for nicotine and coumarin in CYP2A6.17 were 39% and 53% of those in the wild type, respectively. Although Val365 was included in SRS-5, the range of Michaelis constant (*K*_m_) value did not increase in comparison with the wild type [22]. Thus, the V365M mutation was not expected to affect substrate binding in the nicotine–coumarin binding site. Helix C was composed of residues 121–138 in the wild type; however, it was formed by residues 125–134 in CYP2A6.17. In addition, helix L was separated into residues 441–443 and 448–457, and residues 435–437 formed a new helix despite no direct contact with the mutated residue (Table 2). Val365 located on the loop between the helix K and β1-4 interacted with the K”L loop in the wild type, and the distance between the nearest carbon atoms of Val365 and Pro431 was 3.9 Å in the final structure (Figure 7A). On the other hand, the distance between the nearest carbon atoms of Met365 and Pro431 was 8.2 Å in the final structure of CYP2A6.17. In addition, the RMSFs of Pro431–Phe432 were 1.5-fold higher than those of the wild type (Figure 4D). Therefore, the interaction between the K”L loop and the residue 365 decreased and the flexibility of the K”L loop increased in CYP2A6.17. Although the RMSF for other regions of the K”L loop did not increase, a structural change occurred. Residues 434–438 in the K”L loop shifted toward the heme and their percentage of helix formation increased. These shifted residues pushed the heme as shown in Figure 7B, and caused the shift of Ala445, resulting in the separation of helix L. These structural changes around heme and helix L were the cause for the decrease in enzymatic activity of CYP2A6.17. On the other hand, the nicotine and coumarin binding site was on the opposite side of the structurally changed region to the heme. This may be the reason why the V365M mutation had no effect on nicotine and coumarin binding affinity.

### 2.4. CYP2A6.25 and CYP2A6.26 Affected Secondary Structure Formation and Interaction with Heme and Substrates

**Figure 8:**
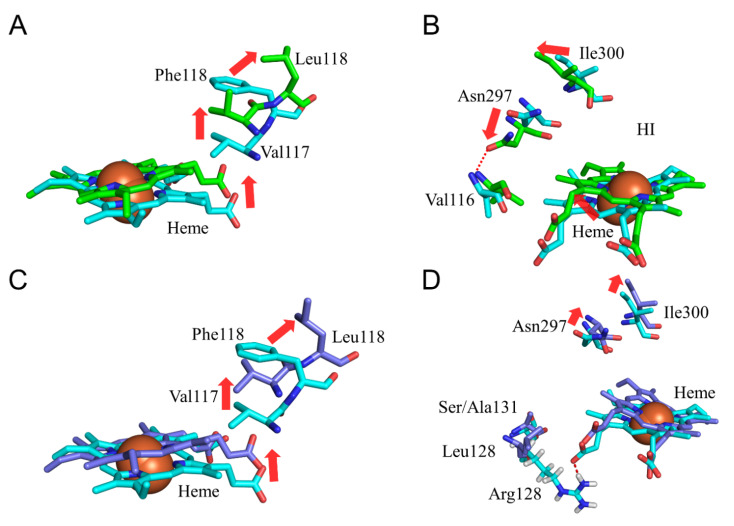
Structural change in CYP2A6.25 and CYP2A6.26 (**A**–**D**). The wild types, CYP2A6.25, and CYP2A6.26 are shown in cyan, green, and marine, respectively. Nitrogen, oxygen, and hydrogen are displayed in blue, red, and white, respectively, in the stick model. Iron is shown as an orange sphere by a model written as van der Waals radius. The red arrows indicate the shift in CYP2A6.25 and CYP2A6.26. The hydrogen atoms are omitted except for Those of Arg128.

To evaluate the structural changes in CYP2A6.25 and CYP2A6.26, the calculated results of F118L and F118L/R128L/S131A mutants were compared with those of the wild type. In CYP2A6.25, *CL_int_* for nicotine was 16% of that of the wild type, and the kinetic parameter for coumarin was not detectable [22]. In CYP2A6.26, the kinetic parameters for both compounds were not detectable [22]. Phe118 was one of the interacting residues with coumarin in the complex structure [15,39], and the adjacent Val117 interacted with nicotine [40]. The location of the side chain of residue 118 moved away from heme in the F118L mutant, and Val117 similarly shifted in CYP2A6.25 (Figure 8A). These alterations were significant in decreasing the binding affinity of nicotine and coumarin. In addition, the location change of heme was caused by the shift of Val117, and the crash of heme with helix I separated the helix into two helices composed of residues 288–296 and 302–319 (Table 2). Due to these alterations, Asn297 and Ile300 moved away from heme, and Asn297 formed an hydrogen bond with Val116 (Figure 8B and Table 6). Asn297 and Ile300 were reported to interact with nicotine and coumarin in the crystal structures of these complexes [15,39]. Therefore, location changes for Val117, Leu118, Asn297, and Ile300 decreased the substrate binding affinity in CYP2A6.25. The deviation of heme might reduce the enzymatic activity of CYP2A6.25. In CYP2A6.26, the shifts of heme and Val117 caused by F118L mutation were also observed (Figure 8C). However, the locations of Asn297 and Ile300 were similar to those of the wild type, and helix I was not separated (Figure 8D). The crash of heme to helix I was weak and had no effect on helix I formation in CYP2A6.26. The loss of the hydrogen bond between Arg128 and heme was caused by R128L mutation and could decrease the binding stability of heme. The crash of heme with helix I could have insufficient influence to separate the helix structure of helix I due to the weakness of the binding stability of heme in CYP2A6.26. In addition, the change in Asn297 location was not observed in this mutant. On the other hand, the structural effects for the S131A mutation were not observed. Therefore, the main factors for the reduction in the activity of CYP2A6.26 could be the decrease in the interaction with heme due to R128L mutation and a decrease in substrate binding affinity by the F118L mutation.

### 2.5. CYP2A6.35, CYP2A6.36, and CYP2A6.37 Affected Interaction with Heme and Substrates

**Figure 9:**
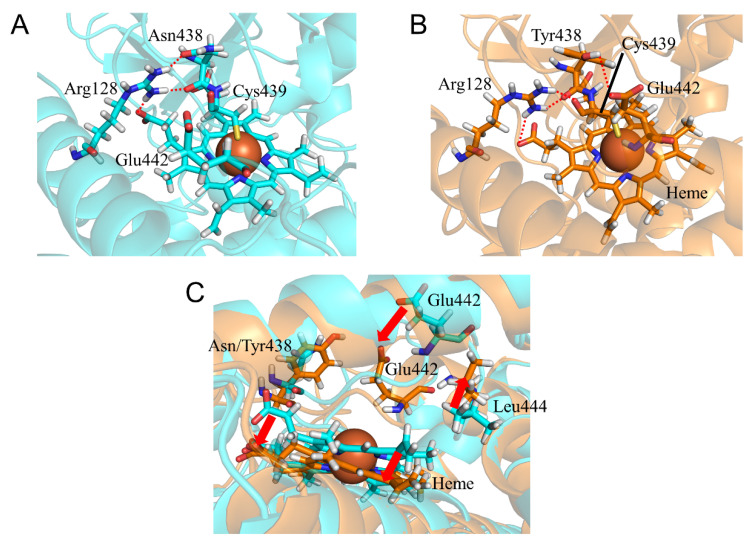
Structural change in CYP2A6.35 (**A**–**C**). The wild type and CYP2A6.35 are shown in cyan and orange, respectively. Nitrogen, oxygen, and hydrogen are displayed in blue, red, and white, respectively, in the stick model. Iron is shown as an orange sphere by a model written as van der Waals radius. The red dotted lines indicate the hydrogen bonds. The red arrows indicate the shift in CYP2A6.35.

**Figure 10:**
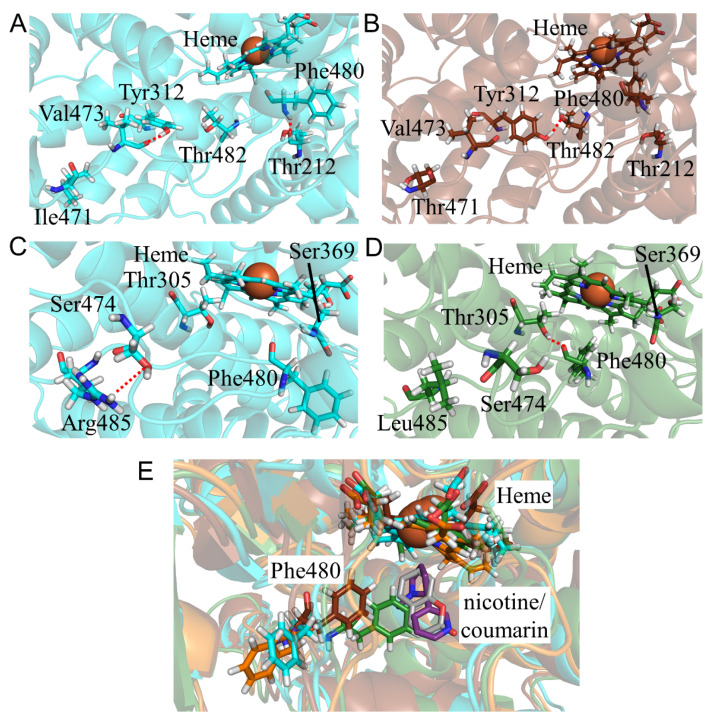
Structural change in CYP2A6.36 and CYP2A6.37 (**A**–**E**). The wild type, CYP2A6.36, and CYP2A6.37 are shown in cyan, deep green and brawn, respectively. Nicotine and coumarin are illustrated in gray and purple, respectively. Nitrogen, oxygen, and hydrogen are displayed in blue, red, and white, respectively, in the stick model. Iron is shown as an orange sphere by a model written as van der Waals radius. The red dotted lines indicate the hydrogen bonds.

To evaluate the structural changes in CYP2A6.35, CYP2A6.36, and CYP2A6.37, the calculated results for N438Y, N438Y/I471T, and N438Y/I471T/R485L mutants were compared with those of the wild type. The CYP2A6.35 had higher *K_m_* and lower *V_max_* values for nicotine and coumarin than those of the wild type, and the *CL_int_* parameters for nicotine and coumarin were 30% and 61% of those in the wild type, respectively [22]. In addition, the kinetic parameters for the CYP2A6.36 and CYP2A6.37 mutants were not detectable. Asn438 is a residue adjacent to Cys439 which is an axial ligand of heme iron. The N438Y mutation affected the hydrogen bond formation around heme. In the wild type, the side- and main-chain oxygen atoms of Asn438 formed hydrogen bonds with Arg128 (Figure 9A and Table 7). In CYP2A6.35, Tyr438 formed an hydrogen bond with Glu442 but the hydrogen bond between the side chain of Tyr438 and Arg128 was not observed (Figure 9B and Table 7). The loss of this hydrogen bond caused interaction changes between the side chain of Arg128 and heme, resulting in the rotation of the carboxylate moiety of heme. In addition, the hydrogen bond formation between Tyr438 and Glu442 caused conformational changes to the K”L loop (Figure 9C). Leu444 was shifted away from heme, and the interaction between Leu444 and heme was attenuated. Those changes in hydrogen bond formation and the K”L loop conformation caused the shift of heme. Therefore, the structural changes around heme were considered the reason for a lower enzymatic activity in CYP2A6.35. Similar structural changes were observed in CYP2A6.36 and CYP2A6.37. In addition, the I471T and R485L mutations affected the structure near the nicotine and coumarin binding sites. In CYP2A6.36, the structural change of the C-terminal region near the mutated residue caused the loss of the hydrogen bond between Tyr312 and Val473, and Tyr312 formed a new hydrogen bond with Thr482 (Figure 10A,B and Table 7). This alteration in hydrogen bond formation shifted the location of Phe480. In CYP2A6.37, the loss of the hydrogen bond formation between Ser474 and Arg485 caused a shift in the location of Phe480 close to heme, and Phe480 formed an hydrogen bond with Thr305 (Figure 10C,D and Table 7). The shifted Phe480 in CYP2A6.36 and CYP2A6.37 invaded the nicotine and coumarin binding sites. Therefore, these structural changes involved in Phe480 were the cause for the decrease in the enzymatic activities of CYP2A6.36 and CYP2A6.37.

### 2.6. CYP2A6.43 Affected the Interaction with Substrates

**Figure 11:**
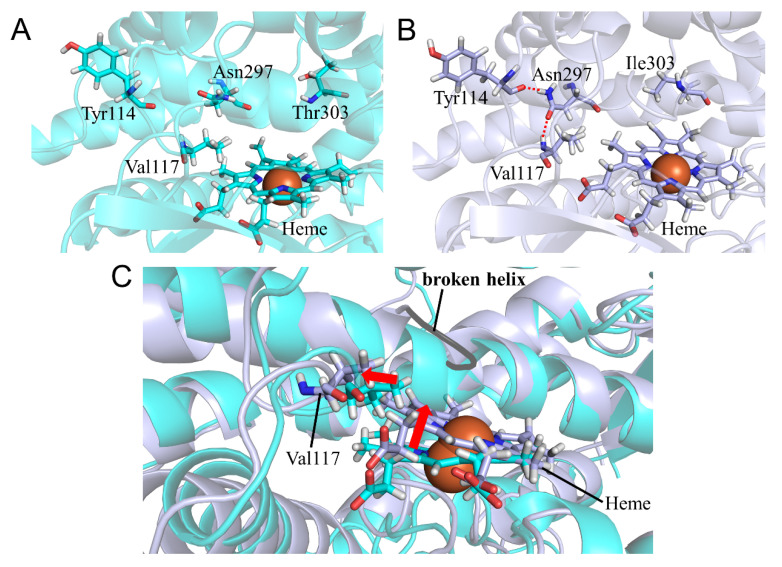
Structural change in CYP2A6.43 (**A**–**C**). The wild type and CYP2A6.43 are shown in cyan and light blue, respectively. Nitrogen, oxygen, and hydrogen are displayed in blue, red, and white, respectively, in the stick model. Iron is shown as an orange sphere by a model written as van der Waals radius. The red dotted lines indicate the hydrogen bonds. The red arrows indicate the shift in CYP2A6.43.

The structural change in CYP2A6.43 was investigated by the comparison between the calculated structures of the wild type and the T303I mutant. In this mutant, the nicotine and coumarin binding affinity for CYP2A6 were much lower than those for wild type [22]. The *CL_int_* for nicotine and coumarin in CYP2A6.43 were 1% and 5% of those in the wild type, respectively. In the calculated results, helix I including the mutated residue was separated into residues 288–297 and 305–319 (Table 3). The helix-broken moiety contained Ile300, which was an important residue for the interaction with nicotine and coumarin [15,39]. In addition, Asn297 shifted away from heme and formed hydrogen bonds with Tyr114 and Val117 (Figure 11A,B and Table 8). These structural changes to helix I were expected to reduce the nicotine and coumarin binding affinity for CYP2A6.43. A shift of heme was also observed and thought to reduce the enzymatic activity. This shift was caused by the structural change of helix I and the shift of Val117. The results indicated that the break of helix I was the main reason for the reduction in the enzymatic activity of CYP2A6.43.

### 2.7. CYP2A6.44 Changed Substrate Access Channel

**Figure 12:**
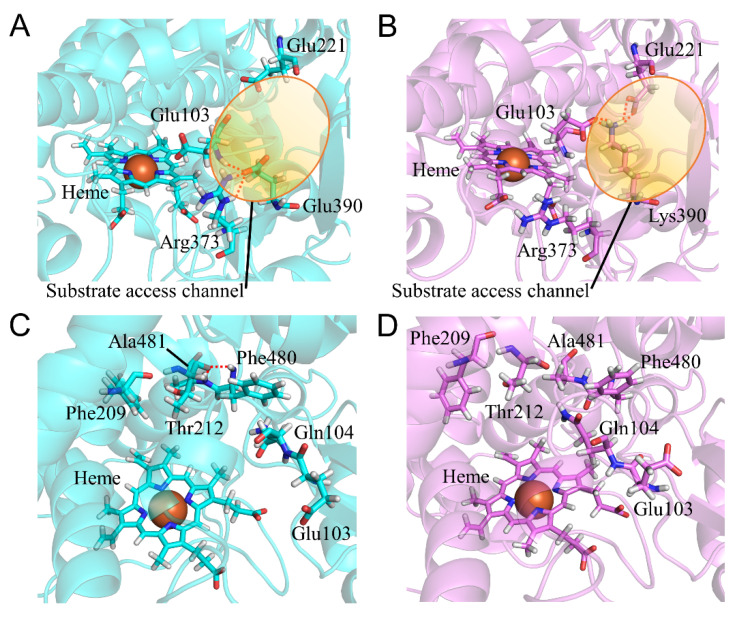
Structural change in CYP2A6.44 (**A**–**D**). The wild type and CYP2A6.44 are shown in cyan and purple, respectively. Nitrogen, oxygen, and hydrogen are displayed in blue, red, and white, respectively, in the stick model. Iron is shown as an orange sphere by a model written as van der Waals radius. The red dotted lines indicate the hydrogen bonds.

To evaluate the structure of CYP2A6.44, we compared the calculated results of the E390K/N418D/E419D mutant with those of the wild type. The *CL_int_* for nicotine was 1% of that in the wild type, and the kinetic parameter for coumarin was not detectable [22]. This mutant had the highest *K_m_* value for nicotine in all of the CYP2A6 mutants. However, the kinetic parameters (*K_m_*, *V_max_*, and *CL_int_*) of the N418D/E419D mutant (CYP2A6.28) were at the same level as that of the wild type. Thus, E390K mutation was assumed to strongly affect substrate binding. Glu390 was located on β1-3 between helices K and K’ and next to the BC loop. β1 and BC loops reportedly composed the substrate access channel [1,41,42,43]. In the wild type, Glu390 formed hydrogen bonds with the main chain of Glu103 and the side chain of Arg373 (Figure 12A and Table 9). In CYP2A6.44, Lys390 formed hydrogen bonds with the main-chain oxygen of Glu103, the side-chain oxygen of Glu103, and the side-chain oxygen of Glu221. The hydrogen bond formation with Glu221 could impair the opening of the substrate access channel. In addition, Glu103 was a residue which formed the substrate access channel and the side-chain atoms of Glu103 in CYP2A6.44, which were in the opposite direction to that in the wild type, located inside of the substrate access channel. The substrate access channel was not seen clearly both in the experimental and calculated structures. Although the difference in the state of substrate access channel was not clearly seen, the E390K mutation was considered to impair access of the substrate to the substrate binding site because of the alteration of the interaction around the substrate access channel. The changes in hydrogen bond formation affected the structures of the substrate binding site. The conformational change of the main chain of Glu103 by the hydrogen bond formation with Lys390 altered the location of Gln104, and the side chain of Gln104 entered the substrate binding site (Figure 12C,D). Displacement of Gln104 triggered a shift in helix F. The hydrogen bonds between Phe480 and Ala481 observed in the wild type were lost in CYP2A6.44 (Table 9). Phe209 moved away from heme. Phe209 interacted with nicotine and coumarin in those complex structures; therefore, the shift of Phe209 reduced the substrate binding affinity for CYP2A6.44 [15,40]. The structural change in the substrate access channel and the substrate binding site could be the key effects of E390K/N418D/E419D mutations on low enzymatic activity. 

## 3. Computational Methods

To construct the initial structures, the experimental structure was retrieved from the protein data bank (PDB). The complex structures of CYP2A6 with nicotine or coumarin were registered (PDB ID: 4EJJ and 1Z10, respectively) [15,40]. Each crystal structure formed tetramers and was devoid of residues 1–29 by the replacement of the N-terminal region for crystallization. Since the resolution and each *R*-value of 1Z10 was better than those of 4EJJ, we used 1Z10 to construct the initial structure. Chain B–C, water molecules, and coumarin were deleted from the experimental structure, and the bond between heme iron and the ligand Cys439 was constructed using AmberTool16. The system was a solvated TIP3P model [44] and was neutralized by adding a counter ion. Structural minimizations of water and counter ion were performed for 1000 steps. Minimizations of the whole system were performed at 2500 steps. Temperature-increasing MD simulations of 20 ps were carried out with the temperature raised from 0 to 300 K, and then, equilibrating MD simulations were performed under constant temperature and pressure. The time step for the MD simulations was 2 fs. The simulation times for the wild type, CYP2A6.6, CYP2A6.36, CYP2A6.37, and CYP2A6.44 were 1000 ns. Other simulation times were extended due to slow convergence. The simulation times for the CYP2A6.25 and CYP2A6.26 were 1200 ns. Those of CYP2A6.11, CYP2A6.17, and CYP2A6.35 were 2000 ns. The cutoff distance for the calculations of the nonbonding interactions was set at 10 Å. The particle mesh Ewald method was used to calculate electrostatic interactions in the periodic boundary condition [45]. The SHAKE algorithm was applied to constrain the lengths of covalent bonds containing hydrogen atoms [46]. All calculations were performed using AMBER16 [47]. Root mean square deviations (RMSDs), RMSFs, and hydrogen bond formations were calculated using the cpptraj module of AmberTools16. The trajectories for the last 10 ns were used in the analysis of RMSF and hydrogen bond formations. AMBER ff14SB force field was used for the amino acid parameters [48]. For heme and the axial ligand Cys439, the parameters determined in a previous study were used [49]. The initial structures of all variants were constructed based on the calculated structure of the wild type after MD simulations.

## 4. Conclusions

We performed the MD simulation for the wild type and 10 mutants and investigated the structural changes related to the reduction in enzymatic activity for the mutants of CYP2A6. In the wild type, the large structural differences in the experimental structure was not observed. The calculated results including heme were available to understand the mechanism for the reduction in the enzymatic activity for the mutants. The effects of the mutations on 3D structures involved in the enzymatic activity reduction are shown in Table 10. The effects of multi mutations were suggested to be additive. The shifts of phenylalanine residues (Phe107, Phe111, Phe118, and Phe480) involved in the interaction with the substrates were crucial for substrate binding. In addition to drug metabolism, CYP2A6 was reported to be involved in hepatocellular carcinoma by contributing to immune regulation [14]. The results of MD simulations could contribute to the prediction of structural changes in CYP2A6 mutants and provide useful information for the relationship between genotype and phenotype. The clarification of the protein–protein interactions between CYP and redox partner is expected to reveal more detail effects of mutations in CYP2A6 variants.

## Figures and Tables

**Table 1:** Kinetic parameters for the wild type (CYP2A6.1) and mutants used in this study. Kinetic parameters for nicotine and coumarin are shown. The percentages of intrinsic clearances (*CL_int_*) are compared with CYP2A6.1 *CL_int._* [22].

		Nicotine				Coumarin	
Protein	Amino Acid Changes	*K_m_*/μM	*V_max_*/pmol min^−1^	*CL_int_*/ nL·min^−1^	*K_m_*/μM	*V_max_*/pmol min^−1^	*CL_int_*/ μL·min^−1^
CYP2A6.1	–	33.3 ± 5.44	5.91 ± 1.26	176 ± 11.2 (100%)	1.94 ± 0.06	5.89 ± 0.34	3.04 ± 0.08 (100%)
CYP2A6.6	R128Q	N.D.	N.D.	N.D.	N.D.	N.D.	N.D.
CYP2A6.11	S224P	142 ± 31.3	4.31 ± 0.34	31.6 ± 5.88 * (18%)	3.30 ± 0.37	2.57 ± 0.03	0.79 ± 0.009 *** (26%)
CYP2A6.17	V365M	25.9 ± 6.16	1.70 ± 0.21	68.3 ± 12.2 * (39%)	1.72 ± 0.03	2.76 ± 0.08	1.60 ± 0.002 ** (53%)
CYP2A6.25	F118L	59.9 ± 5.94	1.67 ± 0.09	28.0 ± 2.48 * (16%)	N.D.	N.D.	N.D.
CYP2A6.26	F118L R128L S131A	N.D.	N.D.	N.D.	N.D.	N.D.	N.D.
CYP2A6.35	N438Y	75.7 ± 14.9	3.98 ± 0.51	53.5 ± 5.31 * (30%)	2.50 ± 0.28	4.63 ± 0.35	1.86 ± 0.08 *** (61%)
CYP2A6.36	N438Y I471T	N.D.	N.D.	N.D.	N.D.	N.D.	N.D.
CYP2A6.37	N438Y I471T R485L	N.D.	N.D.	N.D.	N.D.	N.D.	N.D.
CYP2A6.43	T303I	1049 ± 683	4.76 ± 2.00	5.33 ± 1.28 * (3%)	8.55 ± 1.47	1.34 ± 0.09	0.16 ± 0.02 *** (5%)
CYP2A6.44	E390K N418D E419D	1097 ± 284	2.39 ± 0.43	2.22 ± 0.16 * (1%)	N.D.	N.D.	N.D.

(% of CYP2A6.1), * *p* < 0.05, ** *p* < 0.01, and *** *p* < 0.005 compared with CYP2A6.1. N.D.: Not determined.

**Table 2:** Secondary structure formation in >50% of trajectories for last 100 ns of simulations of the wild type 2A6.6, 2A6.11, 2A6.17, and 2A6.25 ^a^.

	Wild Type	2A6.6	2A6.11	2A6.17	2A6.25
HA	54–65	54–65	54–65	54–65	54–65
β1-1	68–73	68–73	68–73	68–73	68–73
β1-2	76–81	76–81	76–81	76–81	76–81
HB	84–91	84–91	84–91	84–91	84–91
β1-5	100–101	100–101	100–101	100–101	100–101
HB’	105–111	105–108	105–111	105–108	105–111
HC	121–138	122–135	122–138	125–134	121–138
HD	143–163	145–162	143–162	145–160	142–162
HE	171–186	170–186	170–186	170–186	170–186
HF	196–212	196–211	196–212	196–212	196–213
HF’	215–227	215–227	215–222, 224–227	215–227	215–227
HG	233–256	233–257	233–257	233–256	233–256
HH	267–277	267–277	267–277	267–277	267–279
HI	288–319	288–318	288–319	288–319	288–296, 302–319
HJ	321–334	321–334	321–334	321–334	321–334
HJ’	343–347	343–347	343–348	343–348	343–347
HK	350–362	350–362	350–362	350–362	350–362
β1-4	372–373	372–373	372–373	372–373	372–373
β2-1	378–380	378–380	378–380	378–380	378–380
β2-2	383–385	383–385	383–385	383–385	383–385
β1-3	391–393	391–393	390–393	390–393	390–393
HK’	395–399	395–398	395–398	395–398	395–398
HK’’	413–416	413–416	413–416	413–416	413–416
HL	447–459	442–459	441–443, 447–459	435–437, 441–443, 448–457	442–459
β3-1	460–463	460–463	460–463	460–462	460–464
HL’	468–470	468–470	468–470	468–470	468–470
β4-1	477–478	477–478	477–478	477–478	477–478
β4-2	482–483	482–483	482–483	482–483	482–484
β3-2	489–493	489–493	489–493	491–493	489–493

^a^ HX shows helix X, and β shows β-strand formation moiety.

**Table 3:** Secondary structure formation in >50% of trajectories for last 100 ns of simulations of 2A6.26, 2A6.35, 2A6.36, 2A6.37, 2A6.43, and 2A6.44 ^a^.

	2A6.26	2A6.35	2A6.36	2A6.37	2A6.43	2A6.44
HA	54–65	54–65	54–65	54–65	54–65	54–65
β1-1	68–73	68–73	68–73	68–73	68–73	68–73
β1-2	76–81	76–81	76–81	76–81	76–81	76–81
HB	84–91	84–91	84–91	84–91	84–91	84–91
β1-5	100–101	100–101	100–101	100–101	100–101	100–101
HB’	105–110	108–111	105–110	105–111	105–110	107–111
HC	124–138	121–139	121–138	122–137	122–138	121–137
HD	145–162	145–162	143–161	143–161	145–161	143–161
HE	171–186	171–186	171–186	171–186	171–186	171–186
HF	196–212	196–212	196–211	196–213	196–213	196–212
HF’	215–227	215–227	215–227	215–227	215–227	215–227
HG	233–256	233–257	233–256	233–256	233–257	233–256
HH	267–278	267–277	267–278	267–278	267–280	267–277
HI	288–319	288–319	288–319	288–319	288–297, 305–319	288–319
HJ	321–334	321–334	321–334	321–334	321–334	321–334
HJ’	343–347	343–348	343–347	343–347	343–346	343–348
HK	350–363	350–362	350–362	350–362	350–362	350–362
β1-4	372–373	372–373	372–373	372–373	372–373	372–373
β2-1	378–380	378–380	378–380	378–380	378–380	378–380
β2-2	383–385	383–385	383–385	383–385	383–385	383–385
β1-3	390–393	390–393	390–393	390–393	390–393	390–393
HK’	395–398	395–398	395–399	395–398	395–398	395–398
HK’’	413–416	413–416	413–416	413–416	413–416	413–416
HL	442–459	442–459	446–457	447–459	449–457	442–459
β3-1	460–463	460–464	460–464	460–463	460–463	460–463
HL’	468–470	468–470	468–470	470–472	468–470	468–470
β4-1	477–478	–	477–478	477–478	477–478	477–478
β4-2	482–483	–	482–483	482–483	482–483	482–483
β3-2	489–493	489–493	489–493	489–493	489–493	489–493

^a^ HX shows helix X, and β shows β-strand formation moiety.

**Table 4:** Hydrogen bond formation involved in the structural change in CYP2A6.6.

Donor	Donor H	Acceptor	Wild Type	2A6.6
Arg128 N_η1_	Arg128 H_η1_	Heme O_1D_	99.94	0
Arg128 N_η1_	Arg128 H_η1_	Asn438 O	99.92	0
Arg128 N_η2_	Arg128 H_η2_	Asn438 O_δ_	88.72	0
Arg128 N_η2_	Arg128 H_η2_	Asn438 O	33.52	0
Phe429 N	Phe429 H	Ser425 O	0	77.88
Ala428 N	Ala428 H	Ser425 O_γ_	42.66	61.68
His84 N_ε2_	His84 H_ε2_	Asp427 O_δ2_	0	46.76
His84 N_ε2_	His84 H_ε2_	Asp427 O_δ1_	0	38.64
Val430 N	Val430 H	Ala428 O	0	52.86

**Table 5:** Hydrogen bond formation with heme involved in the structural change in CYP2A6.11.

Donor	Donor H	Acceptor	Wild Type	2A6.11
Arg101 N_η1_	Arg101 H_η1_	Heme O_1A_	100	0
Arg101 N_η2_	Arg101 H_η2_	Heme O_1A_	99.98	0.06
Arg101 N_η2_	Arg101 H_η2_	Heme O_2A_	0	98.88
Arg101 N_η1_	Arg101 H_η1_	Heme O_2D_	0	97.48
Arg101 N_η2_	Arg101 H_η2_	Heme O_2D_	0	88.66
Arg101 N_ε_	Arg101 H_ε_	Heme O_2A_	0	65.4
Arg128 N_η1_	Arg128 H_η1_	Heme O_1D_	99.94	81.86
Arg372 N_η2_	Arg372 H_η2_	Heme O_2A_	99.1	48.12
Arg372 N_ε_	Arg372 H_ε_	Heme O_1A_	98.88	74.96
Arg372 N_η2_	Arg372 H_η2_	Heme O_1A_	57.9	0
Arg372 N_ε_	Arg372 H_ε_	Heme O_2A_	51.34	94.66
Ser433 N_γ_	Ser433 H_γ_	Heme O_2A_	100	99.9
Arg437 N_ε_	Arg437 H_ε_	Heme O_2D_	99.32	0.1
Arg437 N_η1_	Arg437 H_η1_	Heme O_2D_	65.76	0.08
Arg437 N_ε_	Arg437 H_ε_	Heme O_1D_	4.5	99.78

**Table 6:** Hydrogen bond formation involved in the structural change in CYP2A6.25 and CYP2A6.26.

Donor	Donor H	Acceptor	Wild Type	2A6.25	2A6.26
Val116 N	Val116 H	Asn297 O_δ1_	0.02	96.38	0
Arg128 N_η1_	Arg128 H_η1_	Heme O_2D_	99.94	99.84	0

**Table 7:** Hydrogen bond formation involved in the structural change in CYP2A6.35, CYP2A6.36, and CYP2A6.37.

Donor H	Donor	Acceptor	Wild Type	2A6.35	2A6.36	2A6.37
Arg128 N_η1_	Arg128 H_η1_	Asn/Tyr438 O	99.92	99.98	83.84	99.78
Arg128 N_η2_	Arg128 H_η2_	Asn/Tyr438 O	88.28	77.68	0.20	97.34
Arg128 N_η2_	Arg128 H_η2_	Asn438 O_δ_/Tyr438 O_η_	33.52	0	7.22	0.02
Arg128 N_η2_	Arg128 H_η2_	Cys439 O	13.86	71.68	99.44	37.70
Arg128 N_η1_	Arg128 H_η1_	Heme O_1D_	99.94	0	0	0
Arg128 N_η1_	Arg128 H_η1_	Heme O_2D_	0	76.96	100	83.90
Tyr312 N_η_	Tyr312 H_η_	Val473 O	58.62	0	0	0
Tyr312 N_η_	Tyr312 H_η_	Thr482 O_γ_	12.90	0	54.78	0
Phe480 N	Phe480 H	Thr212 O	73.92	0	5.10	17.24
Ser369 N	Ser369 H	Phe480 O	39.4	0	28.08	0
Arg485 N_η2_	Arg485 H_η2_	Ser474 O_γ_	69.98	74.78	62.9	–
Arg485 N_ε_	Arg485 H_ε_	Ser474 O_γ_	47.80	20.36	45.84	–
Arg485 N_ε_	Arg485 H_ε_	Ser474 O	32.84	83.22	44.02	–
Thr305 O_γ_	Thr305 H_γ_	Phe480 O	0	0	0	82.26

**Table 8:** Hydrogen bond formation involved in the structural change in CYP2A6.43.

Donor	Donor H	Acceptor	Wild Type	2A6.43
Val117 N	Val117 H	Asn297 O_δ1_	0	98.28
Asn297 N_δ2_	Asn297 H_δ2_	Tyr114 O	0	91.92

**Table 9:** Hydrogen bond formation involved in the structural change in CYP2A6.44.

Donor	Donor H	Acceptor	Wild Type	2A6.44
Glu103 N	Glu103 H	Glu390 O_ε1_	96.67	–
Arg373 N_ε_	Arg373 H_ε_	Glu390 O_ε1_	99.40	–
Arg373 N_η2_	Arg373 H_η2_	Glu390 O_ε1_	95.10	–
Arg373 N_η2_	Arg373 H_η2_	Glu390 O_ε2_	73.22	–
Lys390 N_ζ1_	Lys390 H_ζ1_	Glu103 O_ε1_	–	14.18
Lys390 N_ζ2_	Lys390 H_ζ2_	Glu103 O_ε1_	–	19.30
Lys390 N_ζ3_	Lys390 H_ζ3_	Glu103 O_ε1_	–	35.42
Lys390 N_ζ1_	Lys390 H_ζ1_	Glu103 O_ε2_	–	17.50
Lys390 N_ζ2_	Lys390 H_ζ2_	Glu103 O_ε2_	–	8.72
Lys390 N_ζ3_	Lys390 H_ζ3_	Glu103 O_ε2_	–	6.76
Lys390 N_ζ1_	Lys390 H_ζ1_	Glu103 O	–	19.96
Lys390 N_ζ2_	Lys390 H_ζ2_	Glu103 O	–	32.06
Lys390 N_ζ3_	Lys390 H_ζ3_	Glu103 O	–	22.28
Lys390 N_ζ1_	Lys390 H_ζ1_	Glu221 O_ε1_	–	38.20
Lys390 N_ζ2_	Lys390 H_ζ2_	Glu221 O_ε1_	–	28.04
Lys390 N_ζ3_	Lys390 H_ζ3_	Glu221 O_ε1_	–	25.60
Lys390 N_ζ1_	Lys390 H_ζ1_	Glu221 O_ε2_	–	36.88
Lys390 N_ζ2_	Lys390 H_ζ2_	Glu221 O_ε2_	–	27.58
Lys390 N_ζ3_	Lys390 H_ζ3_	Glu221 O_ε2_	–	25.52
Phe480 N	Phe480 H	Thr212 O	73.92	0
Ala481 N	Ala481 H	Thr212 O	78.72	0

**Table 10:** Structural effects observed in the calculated structures of CYP2A6 mutants.

Protein	Amino Acid Changes	Predicted Structural Effects Involved in the Enzymatic Activity
CYP2A6.6	R128Q	conformational change involved in interaction with the redox partner
CYP2A6.11	S224P	heme deviation, change of secondary structures
CYP2A6.17	V365M	heme deviation, change of secondary structures, conformational change involved in interaction with the redox partner
CYP2A6.25	F118L	structural change of substrate-interacting residues, heme deviation, change of secondary structures
CYP2A6.26	F118L R128L S131A	structural change of substrate-interacting residues, heme deviation, change of secondary structures
CYP2A6.35	N438Y	heme deviation, Cys439 conformational change
CYP2A6.36	N438Y I471T	structural change of substrate-interacting residues, heme deviation, Cys439 conformational change
CYP2A6.37	N438Y I471T R485L	structural change of substrate-interacting residues, heme deviation, Cys439 conformational change
CYP2A6.43	T303I	structural change of substrate-interacting residues
CYP2A6.44	E390K N418D E419D	structural change of substrate access channel, structural change of substrate-interacting residues

## Data Availability

Not applicable.

## Supplementary Materials

The following are available online at https://www.mdpi.com/article/10.3390/ijms221810119/s1.
