# Supplementary material for: Deciphering Structural Alterations Associated with Activity Reductions of Genetic Polymorphisms in Cytochrome P450 2A6 Using Molecular Dynamics Simulations
Source: Int J Mol Sci. 2021 Sep 19;22(18):10119. doi: 10.3390/ijms221810119 (PMC8469730; doi:10.3390/ijms221810119)
Supplement: Supplementary file 1 [file ijms-22-10119-s001.zip › ijms-1366212-supplementary.pdf]

Supplementary Materials

Deciphering structural alterations associated with activity reductions of genetic polymorphisms in cytochrome P450 2A6 using molecular dynamics simulations

Koichi Kato[1,2,3,†], Tomoki Nakayoshi[1,4,†], Rika Nokura[1], Hiroki Hosono,[5,6] Masahiro Hiratsuka[5,6,7,8], Yoshinobu Ishikawa[2], Eiji Kurimoto[1], and Akifumi Oda[1,9,*]

[1] Faculty of Pharmacy, Meijo University, 150 Yagotoyama, Tempaku-ku, Nagoya, Aichi, 468-8503, Japan;
[2] Faculty of Pharmaceutical Sciences, Shonan University of Medical Sciences, 16-48 Kamishinano, Totsuka-ku, Yokohama, Kanagawa, 244-0806, Japan
[3] College of Pharmacy, Kinjo Gakuin University, 2-1723 Omori, Moriyama-ku, Nagoya, Aichi, 463-8521, Japan
[4] Graduate School of Information Sciences, Hiroshima City University, 3-4-1 Ozukahigasi, Asaminami-ku, Hiroshima, Hiroshima, 731-3194, Japan
[5] Graduate School of Pharmaceutical Sciences, Tohoku University, Sendai, Miyagi, 980-8578. Japan
[6] Department of Pharmaceutical Sciences, Tohoku University Hospital, Sendai, Miyagi, 980-8574, Japan
[7] Tohoku Medical Megabank Organization, Tohoku University, Sendai, Miyagi, 980-8573, Japan
[8] Advanced Research Center for Innovations in Next-Generation Medicine, Tohoku University, Sendai, Miyagi, 980-8573, Japan
[9] Institute for Protein Research, Osaka University, 3-2 Yamadaoka, Suita, Osaka, 565-0871, Japan
* Correspondence: oda@meijo-u.ac.jp; Tel.: +81-52-832-1151
† These authors contributed equally to this work.

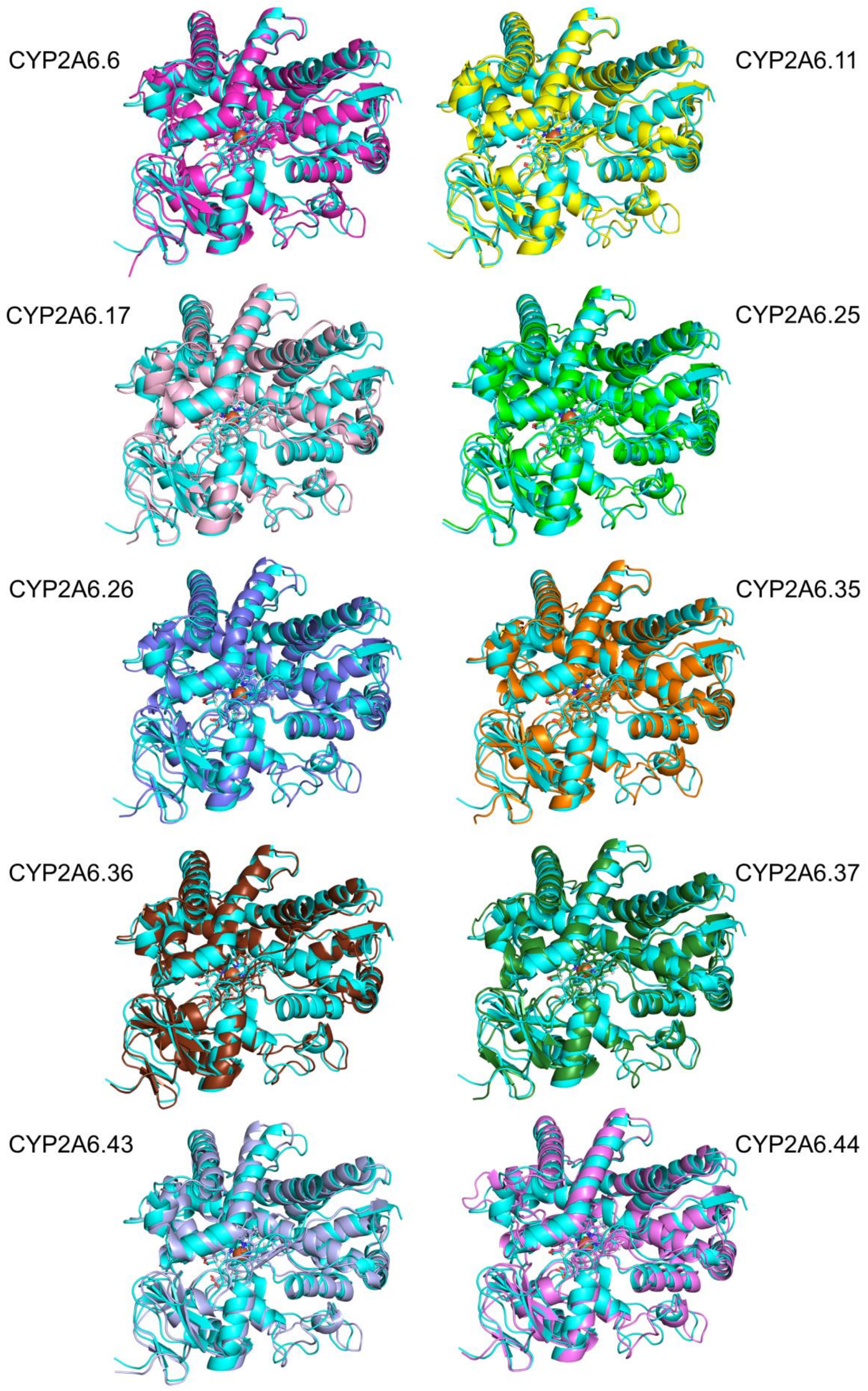

Figure S1. Final structures obtained by MD simulations. All mutant structures are superposed with the simulated structure of the wild type (cyan). Heme and iron are shown in stick and ball models, respectively.
